# Editorial: Immune Dysfunction: An Update of New Immune Cell Subsets and Cytokines in Sepsis

**DOI:** 10.3389/fimmu.2021.822068

**Published:** 2021-12-16

**Authors:** Yong-ming Yao, Marcin F. Osuchowski, Jiang-huai Wang, Zhixing K. Pan

**Affiliations:** ^1^ Translational Medicine Research Center, Medical Innovation Research Division and Fourth Medical Center of the Chinese People’s Liberation Army (PLA) General Hospital, Beijing, China; ^2^ Trauma, Shock and Sepsis Research Group, Ludwig Boltzmann Institute for Experimental and Clinical Traumatology, Vienna, Austria; ^3^ Research Lab, Department of Academic Surgery, National University of Ireland, Cork University Hospital, Cork City, Ireland; ^4^ Department of Medical Microbiology and Immunology, University of Toledo College of Medicine, Toledo, OH, United States

**Keywords:** sepsis, new immune cell subsets, new cytokines, immune dysfunction, novel single-cell RNA-sequencing analysis

The still raging SARS-CoV-2/COVID-19 pandemic has understandably diverted the research attention from other important domains of medical sciences. This Research Topic partly fills the void of the justified neglect and re-focuses the attention on (beyond COVID-19) sepsis – also a globally widespread and burdening problem. Dysregulated host immune response is currently accepted as the major cause of multiple organ dysfunction syndrome (MODS) and subsequent death in septic patients (1, 2). Specifically the dysfunction of innate and adaptive immune systems appears to be responsible for the development of immune deregulation under microbial exposure both regarding the acute sepsis phenotypes as well as their long-term consequences. However, there is currently vague understanding of the pathogenesis of sepsis-induced immune depression, especially of the effects of new immune cell subsets and cytokines.

In recent years, advanced analytical technologies have enabled the discovery of new immune cell subsets and cytokines, which are shown to play various roles in modulating host immune response and initiating MODS secondary to sepsis. This particular Research Topic provides an overview of: i) selected newly discovered immune cell subsets and mediators in the development of sepsis-induced immunosuppression and multiple organ injury, and ii) new functions and modulatory signaling of classical/known immune cells and mediators. The Research Topic features a total of fourteen articles; nine original manuscripts, three review articles, and two mini-reviews. The journal and the Research Topic editors sincerely appreciate all outstanding works and their authors contributing to this collection.

The Sepsis-3 that is defined as life-threatening organ dysfunction caused by a dysregulated host response to infection highlights the importance of immune deregulation in the pathogenesis of sepsis. Darden et al. demonstrated, by using a novel single-cell RNA-sequencing analysis, that the circulating lymphoid cells of late septic patients maintained a transcriptomic profile that was predominantly immunosuppressive with low-grade proinflammatory characteristics. Interestingly, the lymphocyte transcriptomes differed due to various types of infecting organisms, hinting the need for personalized immunotherapy (Darden et al.). While a preliminary report, this analytical direction opens new monitoring frontiers regarding protracted follow-up of chronic sepsis. Using an elegant study-design, Skirecki et al. characterized a compartment-and outcome-dependent evolution of myelopoiesis in acute murine sepsis. Mice with cecal ligation and puncture (CLP) were stratified into predicted-to-die (P-DIE) and predicted to-survive (P-SUR) cohorts revealing a varying rate of depletion of the hematopoietic stem and progenitor cells subclasses depending on the analyzed compartment. These findings implicate the early impairment of myelopoiesis in sepsis outcomes and underline an importance of compartment-targeted monitoring.

Dysregulation of innate and adaptive immune system contributes to sepsis-induced immune deregulation. Several markers have been proposed regarding development of immune dysfunction in sepsis, e.g., a suppressed expression of human leukocyte antigen DR (HLA-DR) on monocytes and lymphocytes. Wang et al. observed a heterogeneous immune response in peripheral blood monocytes of septic patients by using high-dimensional flow cytometry. Furthermore, the authors identified two major monocyte subtypes (i.e., hypo- and hyper-responsive) by assessing the production of cytokines and the HLA-DR expression in response to lipopolysaccharide (LPS) stimulation. Such a diagnostic approach aligns well with the notion of the desired personalized therapy tailored based on immune status. The activation and function of neutrophils in sepsis are another noteworthy topic of this Research Topic. Seree-aphinan et al. screened and compared patients with septic shock versus non-septic infection founding that only C-X-C motif chemokine receptor 2 (CXCR2) was closely associated with the onset of sepsis and concentration-dependent relationship with the clinical severity. Platelet activating factor (PAF) plays an important role in rapid depolarization, intracellular alkalization, and increased cell size of neutrophils. Hug et al. reported that an *ex-vivo* LPS stimulation and porcine polymicrobial sepsis resulted in a diminished response of neutrophils to PAF. This abnormality was recorded at the onset of experimental sepsis as it might serve as an early hallmark of an innate immune dysfunction (Hug et al.). In the next original study, Ji and Fan systematically described a phenomenon of neutrophil reverse migration in sepsis – a potentially one of the major causes of organ injury due to an overexposure of neutrophils. Neutrophils that reversely-migrated from the inflammation/infection sites back into the circulation presented with a prolonged lifespan and delayed apoptosis, showing a positive correlation with sepsis-induced acute lung injury (ALI) (Ji and Fan).

Substantially altered innate immune response frequently has grave short-and long-term consequences. Using a novel technique for intracellular cytokine measurement in the whole blood, Fabri et al. reported the suppressed production of tumor necrosis factor-α (TNF-α) but increased interleukin-10 (IL-10) secretion, and the number of IL-10 producing CD4^+^ T lymphocytes was significantly elevated. An imbalanced responsiveness of both innate and adaptive immune cells undeniably contributes to the overall magnitude of the sepsis-induced immunosuppression. In another mechanistic work, Chen et al. found that activation of group 2 innate lymphoid cells (ILC2s) was able to augment IL-17A-producing γδT cell expansion and secretion of IL-17A by releasing IL-9 in CLP mice. Furthermore, the authors identified the effects of neuromedin U (NMU) on activation of ILC2s by directly binding with NMU receptor 1 (NMUR1), which might be a potential therapeutic target for reversing sepsis-induced ALI. The viability and function of both resident and circulating immune cell subsets are major factors that drive the development of immune dysfunction. Of note, a profound depletion of common myeloid progenitors in P-DIE CLP mice was owing to an increased apoptosis (Skirecki et al.). Other types of cell death, e.g., ferroptosis, could also constitute an essential part of uncontrolled inflammation and immune dysfunction in sepsis (Li et al.). Li et al. systematically reviewed the phenomenon of ferroptosis (characterized by excessive lipid peroxidation and overload ion) and illustrated its close association with a dysregulated immune response. In their pilot study, Lorente-Pozo et al. reported the importance of epigenetic alteration in an impaired immune response in neonatal sepsis. The authors demonstrated that the methylation level in premature septic infants was significant altered in genomic regions functionally associated with multiple inflammatory pathways and innate/adaptive immune responses. These observations suggest that the DNA methylation patterns can be successfully exploited to serve as efficient biomarkers predicting/detecting septic complications.

It is widely accepted that MODS constitutes the ultimate cause of death in septic patients. An excessive inflammation, coagulopathy and/or immune dysregulation are considered as the major mechanisms for the development of multiple organ injury in sepsis. As mentioned above, a delayed withdrawal of neutrophils was critically involved in respiratory tissue damage and the rate of circulating reverse-migrated neutrophils revealed a positive correlation with ALI (Ji and Fan). The IL-17A was observed to be significantly elevated in the lungs of septic mice, urging a further in-depth mechanistic exploration by NMU, ILC2s, and IL-17A-producing γδT cells (Chen et al.). Schulz et al. highlighted the significance of microcirculation and mitochondrial function in the development of gastrointestinal and liver damage during sepsis. The sepsis-induced immune, inflammatory and coagulation fluctuations constitute a complex interwoven network that trigger and drive MODS. Wu et al. discussed the activation of canonical and non-canonical inflammasomes in triggering the release of coagulation factors, such as factor III and tissue factors, from both monocytes and macrophages. This immune-coagulation can be transformed into a vicious cycle for organ damage. Additionally, the authors described a significant role of stimulator of interferon genes (STING) and high mobility group box-1 protein (HMGB1) in modulating the activation of inflammasomes and the immune-to-coagulation interaction (Wu et al.).

An immune suppression does not only contribute to the sepsis onset but it also critically weights upon poor outcomes and protracted post-septic consequences. A timely, efficient and personalized immune-modulation should achieve a high priority in the therapeutic management of sepsis. Bergmann et al. summarized the pathophysiology of the sepsis-induced immunosuppression and highlighted the importance of anti-inflammatory mediators, such as IL-10, transforming growth factor-β (TGF-β), and thymic stromal lymphopoietin (TSLP), in inducing a persistent immune-depression in sepsis. They concluded that modulation of those cytokines should be considered for reversing sepsis-induced immunosuppression once a precise stratification of septic patients is achievable (Bergmann et al.). Likewise, Royster et al. described the role of sialic acid-binding immunoglobulin-type lectin-G (Siglec-G) in immunomodulation by inhibiting activation of nuclear factor κB and damage associated molecular patterns-mediated inflammation. Though greatly desired, any specific drugs for a successful sepsis-induced immunomodulation remain to be added to the current clinical practice. Future pre-and clinical studies should be therefore prioritized on: i) expanding our understanding of the pathophysiological roles of the old and novel immune cell subsets and mediators by using advanced analytical technologies, ii) constructing personalized immuno-therapies based on the patients’ immune status, and iii) combined treatments covering both innate and adaptive immunity (rather than any “golden bullet” therapy) as well as re-balancing the pro- and anti-inflammatory dynamics.

## Author Contributions

All authors contributed to this editorial insight and approved the submitted version.

## Conflict of Interest

The authors declare that the research was conducted in the absence of any commercial or financial relationships that could be construed as a potential conflict of interest.

## Publisher’s Note

All claims expressed in this article are solely those of the authors and do not necessarily represent those of their affiliated organizations, or those of the publisher, the editors and the reviewers. Any product that may be evaluated in this article, or claim that may be made by its manufacturer, is not guaranteed or endorsed by the publisher.
